# The Chronic Migraineur and Health Services: National Survey Results

**Published:** 2015-11-09

**Authors:** Amy Wachholtz, Christopher Malone, Amrita Bhowmick

**Affiliations:** 1 Department of Psychiatry, University of Massachusetts Medical School, Worcester, MA 01501, USA; 2 Health Union, Philadelphia, PA-19113, USA

**Keywords:** Chronic migraine, Headache, Mental health services, Psychiatric symptoms

## Abstract

Chronic migraine is a costly and highly disabling condition that impacts millions of people in the United States. While chronic migraine is hypothesized to result from more infrequent forms of migraine, the precise mechanism by which this develops is still being researched. This study sought to better characterize the treatment patterns, disorder characteristics, and medical and disability profile of the chronic migraine population using the largest dataset of chronic migraineurs ever collected. The survey was started by 8,359 individuals and 4,787 met the inclusion criteria for diagnosed chronic migraine The number of stressful life events participants experienced due to their migraines related to number of therapies tried (p<0.00, eta^2^=0.215), depression (p<0.00, eta^2^=0.178), number of comorbidities (p<0.00, eta^2^=0.172), anxiety (p<0.00, eta^2^=0.162), number of physician visits in the past year (p<0.00, eta^2^=0.103), and chronic pain levels (p<0.00, eta^2^=0.077).. The results of this survey suggest that chronic migraineurs may misattribute aspects of psychiatric or medical comorbidities to their chronic migraines. Further, the sample underutilized mental health services and were unsatisfied with their migraine treatments. Providers to chronic migraineurs should ensure that patients are receiving appropriate mental health care in order to alleviate psychological distress as well as to potentially lessen negative life events previously associated with migraine symptoms.

## Introduction

Migraine headache is a prevalent chronic pain condition that afflicts millions of Americans with prevalence estimates ranging between 16.2% and 22.7% of adults in the United States [1]. Migraine, however, is not a homogeneous disorder but instead is grossly subdivided into two groups: episodic migraine and chronic migraine.

Chronic migraine is the most severe manifestation of migraine and has been found to impart large costs on individuals and society at large with an overall prevalence rate of about 2% [2]. Individuals with chronic migraine have been found to be significantly more disabled than episodic migraineurs with a higher degree of impairment to their daily activities [3,4], have significantly worse medical outcomes, and use healthcare resources at a rate of four times that of episodic migraineurs [5]. Further, chronic migraineurs have been found to experience higher indirect costs of their migraines when compared with episodic migraineurs [6]. Chronic migraineurs have been found to experience lower socioeconomic status and greater psychiatric and medical comorbidities when compared to episodic migraineurs [5].

Chronic migraine is currently differentiated from other forms of migraine based almost exclusively on the frequency of migraine symptoms, however, whether chronic migraine is a distinct entity from other forms of migraine is still debated [5]. Some researchers have argued that migraine is a spectrum of illness with chronic migraine as its most extreme form. This viewpoint is supported by biological research showing that chronic migraine is associated with abnormalities in periaqueductal grey matter damage that may develop progressively in milder forms of migraine [7], both forms show similar patterns of cortical excitability between chronic and episodic migraine [8], and abnormal hypothalamic hormone secretion [9]. Chronic migraine may also be a progression of episodic migraine resulting from medication overuse [10] depression [11], and qualitative disability aspects [12,13]. A smaller number of researchers have argued that chronic migraine is far more distinct from episodic migraine than simple migraine frequency due to distinct biomarkers [14], the unique degradation of the endocannabinoid system in chronic migraine [14,15], different sociodemographic and comorbidity profiles [16], and health quality of life and headache related burden [5].

Whether chronic migraine is an extreme manifestation of the experience of episodic migraine, a distinct neurological or biological entity, or a combination of those etiologies is important, however, research has shown that chronic migraine is understudied and that more information is needed about chronic migraineurs [16]. Knowledge of the comorbid disorders experienced by chronic migraineurs, their treatment patterns, and migraine characteristics, can help inform and improve the treatment satisfaction and care of this group. Previous studies which sought to characterize chronic migraineurs have based their findings on relatively small sample sizes drawn from large survey datasets [17,18]. Chronic migraine is currently thought to be a preventable disorder, so long as appropriate treatment is identified early enough in the development of the disorder and understanding the experience of chronic migraineurs could have vast implication in reducing the individual and societal burdens of chronic migraine [5,19,20].

The personal and societal costs of chronic migraine as well as the previously reported low treatment satisfaction and adherence rates emphasize the need to understand the treatment patterns of this highly disabled population [17]. This need is emphasized with the observation that chronic migraine may progress from or be the result of treatable conditions. The current study extends the available literature by describing the disability profile, migraine characteristics, stress events, treatment patterns, and comorbidity profile of chronic migraineurs. Significantly, the sample reported in this paper is far larger than in any previously reported chronic migraine research.

## Method

### Recruitment

Participants were recruited from a well-known online migraine headache resource. Adults aged 18 years or older and who currently live in the United States were invited to participate. The presence of chronic migraines was validated through agreement with the statement “Have you ever been diagnosed with chronic migraine by a physician?”

### Procedure

This study was approved by the University of Massachusetts Institutional Review Board. An online survey was presented by a migraine-specific community website over 30 days between July and August 2014. Participants were informed that their participation was voluntary, information would be collected anonymously, the anticipated completion time of the survey, and that they would not receive compensation for participating. No personal identifiers were collected nor solicited from participants. The survey contained questions related to demographic information, migraine history and symptoms, social information, and treatment history and satisfaction. The survey host used embedded cookies to prevent a participant from taking the survey multiple times. The survey employed an adaptive survey methodology. Data was collected by the survey host and stored on secure servers.

### Data analysis

All data analysis was performed using SPSS 22.0. Participant demographics were analyzed using descriptive statistics and Analysis of Variance analyses were used to examine the relationships among survey data.

## Results

### Participants

The survey was started by 8,359 individuals. A total of 3,443 individuals were excluded due to: not consenting to participate (n=128), not living in the United States of America (n=677), less than 18 years old (n=69), not currently suffering from migraine (n=57), or not diagnosed with chronic migraine (n=2,449). Of the remaining 4,787 individuals, 3,788 completed it (79.1% completion rate). The mean time to complete the survey was 46.5 minutes (SD=2 hr 0 min 41 sec). Due to the use of adaptive survey methodology, participants did not answer questions that were not relevant to their experience which resulted in a small variation in the number of responses per question.

### Demographics

The sample was 95.1% female and over half of the sample (52.6%) was older than 45 years. Most of the sample (55.8%) had experienced their first migraine symptoms more than 21 years ago and 70.2% had been diagnosed with any type of migraine more than 10 years ago. 33.8% of participants reported being diagnosed with a subtype of migraine and of those individuals, 19.8% reported being diagnosed with Migraine with Aura (ICD-10 G43.1). Additional demographic information can be found in Table 1.

### Comorbid disorders

Data relating to reported comorbid disorders can be seen in Table 2. Over half of the sample had been diagnosed with depression (59.1%) and anxiety (56.4%). The next most frequently reported comorbid disorder was chronic pain at 38.2%. 21.1% of participants reported carrying a diagnosis of chronic fatigue.

### Negative life events

A majority of participants endorsed the following statements describing negative impacts resulting from their migraine: migraines have impacted my work/career (69.2%), people don’t believe that my migraines are severe (64.3%), and constantly worried about disappointing people (53.5%).

A composite variable named “Negative Life Events Score” was created using items from the survey which examined specific areas of life impacted by participant migraines (Cronbach’s alpha=0.837). The Negative Life Events Score was found to have a mean of 4.92 (SD=3.458; Possible Range: 0–13; Observed Range: 0–13). The results of questions assessing the impact of chronic migraine on participant’s lives can be found in Table 3. The results of the ANOVA analyses identifying relationships among the Negative Life Events Score and a variety of migraine and medical aspects can be seen in Table 4. The Negative Life Events Score was found to be account for a high degree of variance in depression (p<0.000, eta^2^=0.1782), anxiety (p<0.000, eta^2^=0.1620), IBS (p<0.000, eta^2^=0.0379), chronic pain (p<0.000, eta^2^=0.0768), the total number of reported comorbidities (p<0.000, eta^2^=0.1717), total number of therapies tried (p<0.000, eta^2^=0.2147), and how many times in the past year that the participant had seen their physician (p<0.000, eta^2^=0.1034).

### Migraine symptoms

**Figure 1:**
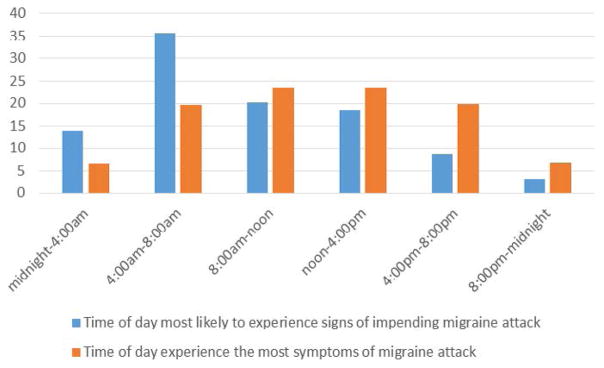
Migraine symptoms and attacks by time of day.

Data relating to migraine symptomology is reported in Tables 5 and 6. Migraine aura was experienced at different rates within the sample with 19.6% of participants reporting that they never experienced aura with their migraines and a further 43.1% of participants reporting that they experienced an aura with their migraines “sometimes”. Head pain was the most commonly endorsed migraine symptom at 84.5% and sensitivity to light was the second most endorsed symptom at 80.1%. Just under half of the sample (45.1%) reported that they currently experience migraine symptoms at a lower frequency than before and of that group only 28.4% attributed finding the right medical approach for the symptom reduction. 48.6% of the sample was able to identify a time of day when they are more likely to experience the signs of an impending migraine attack with 55.8% endorsing the time period of 4:00 am–12:00 pm as the time they are most likely to experience the signs of a migraine attack. Please see Figure 1 for data relating to migraine signs and symptoms by time of day.

### Migraine triggers

82.9% of the sample were able to identify at least one trigger for their migraines. Stress was the most common trigger among this sample at 55.8%. The next most commonly endorsed migraine trigger was lack of sleep at 51.9%. 63.4% of participants had taken active steps to avoid triggers for their migraines. Data relating to migraine triggers can be seen in Table 7.

### Migraine treatments

87.8% of the sample reported consulting a physician for treatment of their migraine and of those 89.3% currently receive treatment from a physician for their migraines. A majority (61.7%) of participants reported disagreeing with a physician about their migraine treatment at some point with the most highly endorsed reason for disagreement being the participant’s previous experience with a treatment that failed (30.5%). 26.1% of participants have used a medication that was not prescribed for their migraine to alleviate their symptoms. A majority of participants have avoided the use of a migraine medication because of its side effects (66.8%) and 75.1% of the sample have discontinued use of a medication because of its side effects. Nearly a third of the sample (30.5%) currently use four or more prescription medications to treat their migraines and 18.9% spend $250 or more per month on these medications and other care for their migraines. Approximately half (48.2%) of the sample used abortive treatment immediately to treat their migraine symptoms; among those who did not use abortive treatment immediately 25.9% did not want to overuse their medication and 22.7% wanted to wait to see if the headache became severe because they did not want to waste a limited supply of medication. Nearly half (48.1%) of the sample “always” incorporates non-pharmacological therapies into their migraine care with dark room being the most used therapy (68.5%). Data relating to physicians consulted about migraine, medical treatments, and therapies, can be seen in Tables 8–10.

### Treatment satisfaction

Chronic migraineur satisfaction with treatment appeared to depend greatly on the aspect of their care that they were being asked about. A majority (62.1%) of the sample was either satisfied or very satisfied with their current physician treating their migraine, however, only 29.4% of the sample was satisfied or very satisfied with their medical treatment for their migraines. 21.9% of participants were satisfied or very satisfied with their non-pharmacological migraine therapy. Data related to treatment satisfaction can be found in Table 11.

### Health care utilization

Nearly half of the sample (48.6%) visited their physician five or more times in the past year specifically for their migraines. 43% of the sample visited an emergency room or urgent care facility at least once in the past year to receive treatment for a migraine, and of that group a third (33.6%) visited the urgent care facility four or more times. Additional information about emergency health care use can be found in Table 12.

### Daily impact

The sample in this study was found to miss an average of 23.5% (14.1 days of previous three months; SD=26.00 days) of their productive time in work or school in the past three months because of their migraines. Their productivity at work or school was reduced by half or more in an additional 29.5% (17.7 days; SD=23.81 days) in the past three months. They reported missing an average of 15.4 (SD=20.75) family, social, or leisure, activities over the past three months due to their headaches. Additional information relating to work and activity impairment due to migraine can be found in Table 13.

## Discussion

The present study characterized the treatment patterns, stress characteristics, disorder characteristics and disability profile, of the chronic migraine population. The sample of this study was predominantly female, however, this is similar to other studies which have found that migraine, and chronic migraine in particular, are much more prevalent in the female population [21]. The sample in the study was demographically similar in age to other cross sectional studies of chronic migraineurs [16]. Similar to previous findings, chronic migraines were found to impart significant financial, medical, and social costs, on individuals.

Chronic migraineurs were found to experience a high degree of disability related to their condition and a low level of satisfaction with regard to their medical and therapeutic approaches despite typically being satisfied with their treating physician. This is significant in suggesting that while chronic migraineurs have typically tried multiple treatments without improvement, they are typically still engaged seeking treatment and have generally not resorted to self-medication. Future research should explore the factors that contribute to the resiliency of chronic migraineurs in the context of high medical disability and chronic pain.

Despite being a cross-sectional study, the data presented in this article suggests that chronic migraine should not be viewed as simply a progression from episodic migraine based on easily quantified factors, such as comorbid medical or psychiatric factors. Previous research has identified depression as a causal factor in the progression of episodic migraine to chronic migraine [11]. The sample in this study had been diagnosed with depression at a lower rate than would have been expected if depression was truly a principal precipitating factor in the chronification of migraine. Similarly, chronic medication overuse has been implicated as a causal factor in the chronification of migraine but the sample in this study was found to be both knowledgeable and cautious in their use of abortive migraine medication. It is possible that the participants in this study were made aware of the dangers of medication overuse only after the development of chronic migraine; providers should emphasize the dangers of medication overuse in the context of the chronification of migraine whenever treating patients with episodic migraine. Additionally, a high proportion of the participants in this study who reported a symptom decrease were ignorant of the cause of the reduction in symptoms, attesting to the heterogeneity of the disorder.

An interesting pattern emerged when migraine symptoms were examined by time of day with many participants endorsing the period between 4:00 am–8:00 am as the period they were most likely to experience the signs of an impending migraine attack. This suggests that the sleep/wake cycle of a migraineur may be clinically significant in the chronification of migraine, however, it may also be that these chronic migraineurs have been misdiagnosed and are instead experiencing hypoxic migraines resulting from undiagnosed or untreated sleep apnea.

Similar to previous findings, the chronic migraineurs in this study endorsed a high degree of negative life events resulting from their migraine with the majority of negative events being experienced as social impairments [22,23]. Significantly, the Negative Life Events Score was found to be highly correlated with a large variety of comorbid disorders but was not significantly correlated with the length of time since first migraine symptoms or the length of time since migraine diagnosis. This suggests that chronic migraine is not a static linear progression from episodic migraine and that it is possible that participants in this sample may attribute impairments due to other comorbid psychiatric and medical disorders to their migraine. In light of the finding that the most common comorbid disorders in this sample could be somaticized psychiatric disorders (i.e. depression and anxiety), it is possible that more serious symptoms of psychiatric disorders are being masked by overlapping migraine symptoms, leading to an underestimate of true psychiatric impairment. Further, the sample in this study utilized mental health services in a far lower degree than would have been expected given their reported psychiatric comorbidities. The sample in this study also reported missing a large number of days of professional, educational, and leisure activities in the previous three months due to their migraine. If even a portion of these days can be attributed to psychological symptoms instead of pure migraine symptoms then it is likely that these participants could be experiencing the symptoms of major mental illness, such as major depressive disorder, further emphasizing the need for psychological intervention.

### Limitations

There are some limitations to the present study. Participants were recruited through an online portal so issues such as self-identification and self-selection cannot be discounted, however, this is common in migraine literature where surveys are the most prevalent method [17]. As discussed previously, the gender composition of this sample was slightly more female than samples in other large chronic migraine samples [19]. The lack of information collected relating to participant race, education, or socioeconomic status may also limit the generalizability of this study.

## Conclusion

The present study extends the literature characterizing the treatment patterns, stress characteristics, disorder characteristics and disability profile, of the chronic migraine population in the largest sample yet collected. Chronic migraineurs were found to experience significant impairment to their personal, professional, and social functioning. Psychological comorbidities were suggested to exert a magnifying effect on the negative experiences of migraine headache with a corresponding underutilization of mental health interventions in this sample. Chronic migraineurs were found to have tried many different treatments but are, overall, unsatisfied with their efficacy while simultaneously being satisfied with their treatment provider. Future research should explore the role of psychiatric distress and somatization as a causal factor in the chronification of migraine. Current providers of chronic migraineurs should emphasize the importance of psychological care in the context of a chronic illness to both address the common psychological component as well as to potentially lessen the impact of chronic migraine on daily life.

## Figures and Tables

**Table 1:** Patient demographics and disease characteristics.

	N	%
Gender		
Male	233	4.9
Female	4554	95.1
Age in Years		
<18	69	(Excl)
18–24	258	3.2
25–34	1054	13.2
35–44	2471	30.9
45–54	2405	30.1
55–64	1416	17.7
>65	390	4.8
First Migraine Symptoms		
<1 year	28	0.6
1–5 years	343	7.3
6–10 years	479	10.0
11–15 years	588	12.3
16–20 years	672	14.0
21 or more years	2671	55.8
US Resident		
Live in USA or its territories	7386	100
US citizen living abroad	167	(Excl)
Do not live in USA and not a US citizen	510	(Excl)
Ever been diagnosed with chronic migraine by physician		
Yes	4619	100
No	2449	(Excl)
Diagnosed with another type of migraine at any point		
Yes	1618	33.8
No	1782	37.2
Not sure	1387	29.0
What other type of migraine were you diagnosed with? (ICD-10 code)		
Episodic Migraine (G43.909)	892	18.6
Migraine without Aura (G43.00)	655	13.7
Menstrual Migraine (G43.82)	533	11.1
Migraine With Aura (G43.1)	947	19.8
Migraine with Typical Aura (G43.109)	159	3.3
Migraine with Brainstem Aura (G43.109)	96	2.0
Hemiplegic Migraine (G43.4)	243	5.1
Retinal Migraine (G43.1)	200	4.2
Abdominal Migraine (G43.D)	116	2.4

**Table 2:** Comorbid disorders.

Comorbidity	N	(%)
Depression	2830	59.1
Anxiety	2699	56.4
Stroke	145	3.0
Epilepsy	93	1.9
Hypertension	972	20.3
Diabetes	250	5.2
Bipolar Disorder	253	5.3
IBS	1282	26.8
Crohn’s Disease	44	0.9
Cardiovascular disease	87	1.8
High Cholesterol	920	19.2
Chronic Pain	1827	38.2
Fibromyalgia	847	17.7
Thyroid Disease	784	16.4
Chronic Fatigue	1010	21.1
Asthma	763	15.9
COPD	87	1.8
Hepatitis	41	0.9
Sleep Disorders	1447	30.2
Rheumatoid Arthritis	182	3.8
Multiple Sclerosis	21	0.4
Autoimmune Disorder	218	4.6
PTSD	497	10.4
OCD	318	6.6
Panic Disorder	643	13.4
Phobia	185	3.9
Borderline-Personality Disorder	74	1.5
Substance Abuse	136	2.8
Pancreatitis	62	1.3

**Table 3:** Negative life events.

Event		N	%
People don’t believe that my migraines are severe		3080	64.3
I’m constantly worried about disappointing people		2559	53.5
Migraines have impacted my work/career		3313	69.2
Migraines affect my ability to maintain relationships		2036	42.5
Migraines impact my relationship with my child/children		1890	39.5
I feel like others blame me for my migraines		1757	36.7
Sometimes I blame myself for my migraines		1467	30.6
Friends/family/colleagues treat me differently because of my migraines		1888	39.4
Doctors treat me differently because of my migraines		1418	29.6
I feel embarrassed about having migraines		1449	30.3
I have lost a job due to migraines		1241	25.9
I’ve lost friends due to my migraines		1086	22.7
Migraines contributed to my divorce/separation		351	7.3
	**Mean**	**SD**	**Range**
Negative life events score total score	4.92	3.458	0–13

**Table 4:** ANOVA analysis of negative life events score triad by comorbid disorders.

	F(df-btw, df-tot)	p	Eta^2^
Depression	207.337(13, 4786)	0.000	0.1782
Anxiety	184.854(13, 4786)	0.000	0.1620
Stroke	3.484(13, 4786)	0.002	0.0036
Epilepsy	1.861(13, 4786)	0.099	0.0019
Hypertension	16.967(13, 4786)	0.000	0.0174
Diabetes	5.084(13, 4786)	0.000	0.0053
Bipolar	10.781(13, 4786)	0.000	0.0112
IBS	37.635(13, 4786)	0.000	0.0379
Crohn’s Disease	2.505(13, 4786)	0.018	0.0026
Cardiovascular Disease	3.049(13, 4786)	0.010	0.0032
High Cholesterol	13.037(13, 4786)	0.000	0.0135
Chronic Pain	79.545(13, 4786)	0.000	0.0768
Fibromyalgia	18.310(13, 4786)	0.000	0.0188
Thyroid Disease	14.204(13, 4786)	0.000	0.0146
Chronic Fatigue	33.658(13, 4786)	0.000	0.0340
Asthma	13.070(13, 4786)	0.000	0.0135
COPD	1.992(13, 4786)	0.026	0.0021
Hepatitis	1.092(13, 4786)	0.273	0.0011
Sleep Disorders	50.430(13, 4786)	0.000	0.0501
Rheumatoid Arthritis	5.370(13, 4786)	0.003	0.0056
Multiple Sclerosis	2.781(13, 4786)	0.846	0.0029
Autoimmune Disorder	4.672(13, 4786)	0.000	0.0049
PTSD	22.716(13, 4786)	0.000	0.0232
OCD	15.546(13, 4786)	0.000	0.0150
Panic Disorder	37.606(13, 4786)	0.000	0.0378
Phobia	13.026(13, 4786)	0.000	0.0134
Borderline Personality Disorder	2.732(13, 4786)	0.000	0.0029
Substance Abuse	8.240(13, 4786)	0.000	0.0085
Pancreatitis	1.890(13, 4786)	0.115	0.0020
Total Number of Reported Comorbidities	198.156 (5, 4786)	0.000	0.1717
Total Number of Therapies Tried	261.405(5, 47860)	0.000	0.2147
Age	13.679 (13, 4786)	0.000	0.0359
How long ago did you first start experiencing migraine symptoms?	1.273 (13, 4786)	0.221	0.0035
How long ago were you first diagnosed with migraine?	0.614 (13, 4786)	0.845	0.0017
How many times in the past year would you estimate that you have been to see your physician?	37.933 (13, 4290)	0.000	0.1034
How many times in the past year have you been to an emergency room or urgent care facility for your headache/migraine?	14.592 (13, 4290)	0.325	0.0425

**Table 5:** Migraine symptoms.

Symptoms	N	%
Do you experience an aura with your migraines?		
Always	544	13.1
Most Times	1000	24.2
Some Times	1784	43.1
Never	809	19.6
Head pain	4045	84.5
Sensitivity to light	3832	80.1
Nausea/vomiting	3384	70.7
Diarrhea/constipation	1342	28.0
Diff Concentrating	3420	71.4
Fatigue	3232	67.5
Neck pain	3076	64.3
Dizziness/lightheadedness	2588	54.1
Sensitivity to sound	3555	74.3
Visual Changes	2453	51.2
Weakness	1984	41.4
Mood Changes	2554	53.4
Sensitivity to Smell	2868	59.9
Numbness/tingling	1522	31.8
Vertigo	1425	29.8
Puffy Eyelid	1067	22.3
Food Craving	1001	20.9
Other	781	16.3

**Table 6:** Reasons for decreases in migraine symptom frequency.

	N	%
Currently Experience the highest level of migraine symptoms	2615	54.6
Reasons for symptom decrease		
Symptoms decreased on their own	160	3.3
Symptoms decreased because of right non-pharmacological approach	561	11.7
Found the right medicinal approach	1360	28.4
Found the right doctor	809	16.9
Found out how to avoid specific	1068	22.3
Decreased as I got older	210	4.4
Psycho-social approach to pain	77	1.6
Psychotherapy	81	1.7
Stress management	559	11.7
Symptoms decreased due to another reason	522	10.9

**Table 7:** Migraine triggers.

Migraine Triggers	N	%
Identified any Triggers	3416	82.9
Stress	2669	55.8
Environment (weather, etc…)	2870	60.0
Lack of Sleep	2484	51.9
Hormones/menstrual cycle	1792	37.4
Certain food drink	2138	44.7
Missing meals	1884	39.4
Certain smell	2046	42.7
Alcohol/drugs	1235	25.8
Physical Activity	1241	25.9
Sexual Activity	325	6.8
Other	739	15.4
Steps taken to avoid triggers	3033	63.4

**Table 8:** Physicians consulted about migraine.

	N		%	
Do you currently see a physician for migraine	3749		89.3	
Ever disagreed with a physician about migraine treatment	2307		61.7	
Meds not strong enough	799		16.7	
Previous experience with treatment failed	1459		30.5	
Demanded too much time/energy to follow through	182		3.8	
Not covered by insurance	441		9.2	
Other reason	825		17.2	
Reasons a Physician is not Consulted				
Inconvenience/time issues	18		4.0	
Financial/Cost/Insurance issues	162		35.8	
Can’t find the right doctor	80		17.7	
It never occurred to me	7		0.1	
Other	185		40.9	
Physicians	Physicians Ever Consulted		Physicians Currently Treating	
	N	%	N	%
PCP/internist	3875	80.9	1798	37.6
Ob/Gyn	1159	24.2	122	2.5
Neurologist	3741	78.1	2274	47.5
Headache Specialist	2003	41.8	992	20.7
Other physician	814	17.0	369	7.7

**Table 9:** Medication and illicit substance use.

	N	%
How quickly do you initiate abortive treatment to stop and/or to treat symptoms?		
Immediately	2046	48.2
Within one hour	1368	32.2
One to two hours	410	9.7
Two to four hours	157	3.7
More than four hours	37	0.9
N/A	227	5.3
Reasons not treated immediately		
I need to make sure it is really a migraine	899	18.8
Don’t want to waste medication	1086	22.7
I get a lot of false alarms	114	2.4
I don’t want to overuse medications	1239	25.9
Other	330	6.9
Illicit Treatments		
Used a drug that was not prescribed to treat migraine	1056	26.1
Ever used illicit drug to treat migraine	385	9.5
Used marijuana	806	19.9
Used alcohol to treat migraine	289	7.1
Used nicotine/tobacco to treat migraine	189	4.7
Ever avoided a medication because of side effects	2699	66.8
Ever stopped a medication due to side effects	3034	75.1
Stopped due to:		
nausea/vomiting	1075	22.5
stomach ache	600	12.5
rebound headaches	1378	28.8
dizziness	966	20.2
cognitive challenges	1677	35.0
other reason	1687	35.2
Avoided medicine due to cost	1961	48.9
Withheld medication because you wanted to “spare” or “save” medication	3055	76.1
How many different *prescription* products do you currently use to treat migraine		
0	295	7.4
1	662	16.6
2	969	24.3
3	839	21.1
4 or more	1215	30.5
How many (if any) *prescription* products have you EVER USED to TREAT your migraines? By “ever used” we mean that you have ever taken at any point to treat your migraines.		
0	23	0.6
1	108	2.7
2	289	7.3
3	415	10.4
4	412	10.4
5	311	7.8
6 or more	2422	60.9
How much would you estimate that you pay out of pocket monthly for medications and care related to your migraines, including insurance co-pays?		
0–100	1824	45.5
101–250	1135	28.3
251–500	469	11.7
>500	288	7.2
not sure	297	7.4

**Table 10:** Non-prescription therapies.

	Therapies tried		Currently Use therapies	
	N	%	N	%
Acupuncture	1386	29.0	241	5.0
Dark room	3672	76.7	3277	68.5
Diet	2634	55.0	1809	37.8
hot/cold therapy	3223	67.3	2677	55.9
Magnesium	2387	49.9	1353	28.3
Riboflavin/B2	1511	31.6	743	15.5
Psychotherapy/pain psychologist	800	16.7	245	5.1
Biofeedback/EEG/EMG	932	19.5	176	3.7
Transcranial Magnetic Stimulation	204	4.3	70	1.5
Light Therapy	265	5.5	102	2.1
Herbals	1570	32.8	616	12.9
Movement Therapy-PT/Yoga…	1024	21.4	415	8.7
Massage	2488	52.0	1244	26.0
Energy Therapy - Reiki	365	7.6	99	2.1
Spiritual/religious resources	744	15.5	515	10.8
other	807	16.9	598	12.5
How often are non-medicinal therapies used				
	N		%	
never	279		7.2	
rarely	198		5.1	
half the time	501		13.0	
most times	1024		26.5	
always	1855		48.1	

**Table 11:** Treatment satisfaction.

	Treatment Satisfaction		How satisfied are you with your current non-prescription therapies for migraine		How satisfied with current physician	
	N	%	N	%	N	%
Extremely Satisfied	182	4.5	101	2.6	1064	28.5
Satisfied	1010	24.9	757	19.3	1256	33.6
Neutral	1426	35.2	1777	45.4	947	25.3
Dissatisfied	878	21.7	820	21.0	333	8.9
Extremely Dissatisfied	523	12.9	328	8.4	138	3.7
Not applicable	35	0.9	130	3.3	450	9.7

**Table 12:** Emergency healthcare.

How many times in the past year would you estimate that you have been to see your physician (not counting any emergency room visits) regarding your headache/migraine?		
	N	%
1 or less	549	12.8
2	493	11.5
3	495	11.5
4	668	15.6
5 or more	2068	48.6
Have you been to an emergency room or urgent care facility in the past year for your headache/migraine?		
	N	%
Yes	2057	43.0
How many times in the past year have you been to an emergency room or urgent care facility for your headache/migraine?		
	N	%
1	547	26.6
2	495	24.1
3	321	15.6
4 or more	690	33.6

**Table 13:** Activity impairment.

	Mean	SD	% of Days Impacted
On how many days in the last 3 months did you miss *work or school* because of your headaches?	14.1	26.00	23.5
How many days in the last 3 months was your productivity at *work or school* reduced by half or more because of your headaches? (Do not include days you counted in the previous question where you missed work or school entirely.)	17.7	23.81	29.5
On how many days in the last 3 months did you not do household work (such as housework, home repairs and maintenance, shopping, caring for children and relatives) because of your headaches?	24.2	22.77	26.9
How many days in the last 3 months was your productivity in household work reduced by half of more because of your headaches? (Do not include days you counted in the previous question where you did not do household work.)	21.1	21.28	23.4
On how many days in the last 3 months did you miss family, social or leisure activities because of your headaches?	15.4	20.75	17.1
How many times in the past year would you estimate that you have been to see your physician (not counting any emergency room visits) regarding your headache/migraine?	3.8	1.47	
